# Others’ emotions teach, but not in autism: an eye-tracking pupillometry study

**DOI:** 10.1186/s13229-016-0098-4

**Published:** 2016-08-30

**Authors:** Heather J. Nuske, Giacomo Vivanti, Cheryl Dissanayake

**Affiliations:** 1 Center for Mental Health Policy and Services Research, Perelman School of Medicine, Department of Psychiatry, University of Pennsylvania, Philadelphia, PA 19104 USA; 2 Olga Tennison Autism Research Centre, School of Psychological Science, La Trobe University, Melbourne, Victoria 3086 Australia; 3 A. J. Drexel Autism Institute, Drexel University, Philadelphia, PA 19104 USA

**Keywords:** Social-emotional calibration, Autism, Emotion, Eye-tracking pupillometry, Autonomic nervous system, Social learning

## Abstract

**Background:**

Much research has investigated deficit in emotional reactivity to others in people with autism, but scant attention has been paid to how this deficit affects their own reactions to features of their environment (objects, events, practices, etc.). The present study presents a preliminary analysis on whether calibrating one’s own emotional reactions to others’ emotional reactions about features of the world, a process we term *social-emotional calibration*, is disrupted in autism.

**Methods:**

To examine this process, we used a novel eye-tracking pupillometry paradigm in which we showed 20 preschoolers with autism and 20 matched typically developing preschoolers’ videos of an actor opening a box and reacting to the occluded object inside, with fear or happiness. We expected preschoolers to come to perceive the box as containing a positive or threatening stimulus through emotionally calibrating to the actor’s emotional expressions. Children’s mean pupil diameter (indicating emotional reactivity) was measured whilst viewing an up-close, visually identical image of the box before and then after the scene, and this difference was taken as an index of social-emotional calibration and compared between groups.

**Results:**

Whilst the typically developing preschoolers responded more emotionally to the box after, compared to before the scene (as indexed by an increase in pupil size), those with autism did not, suggesting their reaction to the object was not affected by the actor’s emotional expressions. The groups did not differ in looking duration to the emotional expressions; thus, the pupil dilation findings cannot be explained by differences in visual attention. More social-emotional calibration on the happy condition was associated with less severe autism symptoms.

**Conclusions:**

Through the measurement of physiological reactivity, findings suggest social-emotional calibration is diminished in children with autism, with calibration to others’ positive emotions as particularly important. This study highlights a possible mechanism by which individuals with autism develop idiosyncratic reactions to features of their environment, which is likely to impact their active and harmonious participation on social and cultural practices from infancy, throughout the lifespan. More research is needed to examine the mediators and developmental sequence of this tendency to emotionally calibrate to others’ feelings about the world.

## Background

People with Autism Spectrum Disorder (ASD), a neurodevelopmental disorder characterised by impairments in social communication and behavioural rigidity [1], often present with atypical, reduced or delayed reactions to others’ emotions [2, 3]. They also frequently present with idiosyncratic, extreme or otherwise unusual emotional reactions to particular features in their physical environment (objects, sensations, events, etc.) [4–7]. Though these phenomena are often documented in ASD, the link between atypical emotional responses to people and atypical emotional responses to non-social environmental features is not clear. In particular, no study to date has investigated whether observing another person reacting emotionally to features of the world influences how individuals with ASD react to those features, for example, how a teacher smiling while reading a book influences the reaction of a boy with autism to that book.

We know that emotional expressions of significant others teach young typically developing children about the world around them, without the need of explicit verbal instruction; by 12 months, children spontaneously seek out emotional feedback from their caregivers to determine appropriate behavioural adjustments in different contexts (i.e. they engage in *social referencing*; [8–12]). Acquiring knowledge about how people react to features of the world from others’ emotions is an effective and efficient learning mechanism that children use prior to language onset and which continues to shape behaviour throughout the lifespan [13].

Preliminary research on such learning from others’ emotions in ASD suggests difficulties in this area relative to their typically developing peers [14, 15], which is keeping with our current understanding of ASD as a disorder of *social learning* [16–18], i.e. learning from other people. However, given that reduced/atypical attention to social stimuli, such as faces, is a common feature of ASD [19, 20], it is unclear whether these difficulties are the consequence of not seeking out or paying attention to others’ emotional expressions regarding features of their environment, or whether children with ASD do look but fail to calibrate their own emotional reactions to the reactions of others. We define this process, *social-emotional calibration*, a process by which, after observing or experiencing another’s emotional expression (e.g. facial, vocal or bodily) in response to a particular referent (e.g. an object, event, topic, social or cultural practice, attitude or another person), the observer’s emotional reactions to that referent calibrate with those of the observed person. It is proposed that children learn to react appropriately to objects and situations in their environment based on the observed emotions of others toward those objects or situations. For example, through seeing his mother laugh at a clown, a child can learn that the clown is not scary and join in laughing at it. Likewise, by observing the pleasure expressed on other children’s faces at birthday parties, a child can be moved to enjoy the event and look forward to his upcoming birthday.

Like social referencing, social-emotional calibration is a triadic process between two people and a referent. However, whilst some previous work using social referencing paradigms has investigated the act of *seeking out* others’ emotional reactions to guide behaviour, the current study focuses on the *change* in emotional reaction to a particular referent that occurs as a consequence of witnessing others’ emotions to that referent. The foundation of this process, we argue, is rooted in early social attention and social-emotional processes including gaze following, emotion recognition and emotional contagion [21, 22]. Individuals with ASD have difficulties with each of these processes, including reduced attention to people and difficulties in recognising and implicitly responding to others’ emotions and expressing their own emotions, leading to difficulties in empathic responding in this population [3]. A large body of research has investigated physiological and neurophysiological reactivity to others’ emotions in ASD. Findings suggest atypical responses to both positive and negative emotions at both levels of implicit and explicit processing (e.g. [23–30]). Furthermore, slower responses to emotions have been reported across physiological and behavioural methodologies [31–35]. Therefore, we expected children with ASD to have reduced social-emotional calibration due to difficulties with processing others’ emotions. We wanted to know whether children with ASD learn to react appropriately to objects and situations in their environment based on the observed emotions of others to those objects and situations.

To date, most of the research referred to above has investigating physiological and neurophysiological emotional reactivity has focused on older high-functioning children and adults due to the invasive and movement-sensitive technology commonly used in these studies (event-related potentials (ERP), functional magnetic resonance imaging (fMRI), electrocardiogram (ECG)). Little is known about emotion processing in younger, more affected children, and the findings mentioned above may not apply to this population. Eye-tracking pupillometry is an ideal measure of physiological responses in this group as no electrodes are required for recording and this technology is more tolerant of movement than other physiological recording systems (e.g. systems for measuring skin conductance responses). Pupillometry measures pupil size over time; the pupils dilate in response to stimuli that are high in emotional intensity, regardless of valence (e.g. [36–39]). Pupil dilation has been found to be correlated with skin conductance responses whilst viewing emotional images [36], which is indicative of the role of sympathetic nervous system responses. Atypical pupil dilation to emotion-inducing stimuli has been found in clinical populations such as people with depression [40, 41] and post-traumatic stress disorder [42], as well as children at risk for depression and anxiety [43] and healthy adults who have been sleep-deprived [44]. Recently, this technology has been applied to individuals with ASD to look at responses to social vs. non-social stimuli in lower-functioning young children [45]. Since then, pupillometry has been used to examine a number of questions in ASD (and children at risk for ASD) including those related to resting-state physiology [46–48], sensory-processing [49, 50], eye-gaze processing [51] and general face processing [52]. Pupillometry findings on emotion processing in ASD are consistent with findings from on older, more-able children and adults, including finding reduced and delayed emotional responses in the population [24, 30, 53–55].

### The current study

Our primary aim was to test the hypothesis that social-emotional calibration is disrupted in children with ASD, compared to typically developing children. Additionally, we wanted to understand whether difficulties in calibrating to others’ emotions reflect a lack of attention to others’ emotional expressions, or difficulties in social-emotional calibration per se, and whether ASD symptoms are associated with social-emotional calibration.

## Methods

### Participants

Twenty-six children with ASD and 24 typically developing (TD) children, aged 2 to 5 years, participated in the study. However, six children in the ASD group and four children in the TD group were excluded as they looked <20 % of the duration of one or both of the up-close images of the boxes shown in the video stimuli (pre-/post-box, see ‘Materials’ section), resulting in a total of 20 children in each group. This threshold was chosen as this allowed us to obtain a reasonable duration of minimum viewing time (800 ms) in order to determine the mean pupil diameter whilst viewing the boxes. Participant characteristics are presented in Table 1. Both groups were recruited through the same community childcare centre offering services for children with ASD and TD children. The Mullen Scales of Early Learning (MSEL; [56]) was administered to all participants to measure cognitive ability. As expected, the ASD group was lower in cognitive ability than the TD group. Cognitive ability was entered as a covariate in the analysis. Following the recommendations of Dykens and Lense [57], the sample included low- to high-functioning children with ASD [58], with 65 % low functioning (standard score <70), 10 % moderately functioning (standard score 70–84) and 25 % high functioning (standard score ≥85).

**Table 1:** Participant characteristics

	ASD group (*N* = 20)	TD group (*N* = 20)	Comparison coefficients
Age (years): *M* (SD)	4.03 (1.09)	4.25 (.63)	*t*(38) = .75, *p* = .46
Gender: M, F	16, 4	16, 4	–
MSEL, SS: *M* (SD)^a^	69.50 (23.10)	102.00 (17.84)	*t*(38) = 4.98, *p* < .001
ADOS, C-SevSc: *M* (SD)^b^	6.45 (2.44)	–	–
ADOS, C-SA: *M* (SD)^c^	11.80 (4.14)	–	–
ADOS, C-RRB: *M* (SD)^d^	4.40 (2.14)	–	–

^a^Mullen Scales of Early Learning Standard Score (Early Learning Composite)

^b^Autism Diagnostic Observation Schedule: Calibrated Severity Score

^c^Autism Diagnostic Observation Schedule: Calibrated Social Affect Score

^d^Autism Diagnostic Observation Schedule: Calibrated Restricted and Repetitive Behaviours Score

Clinic-based diagnoses of the children with ASD were confirmed using the Autism Diagnostic Observation Schedule [59], by clinicians certified for research purposes on the administration and coding of the Autism Diagnostic Observation Schedule (ADOS), with 14 children meeting the more strict cut-off for autistic disorder and 6 meeting criteria for ASD. The ADOS scores were converted to ADOS calibrated severity scores using the algorithms provided by Gotham et al. [60]. One participant was taking methylphenidate at the time of testing. However, as this participant was not an outlier on any dependent variable, and given that results remained unchanged with the exclusion of his data, he was retained in the sample. All participants were free from any other medical conditions, and had no visual, hearing or motor impairments. The research was approved by the La Trobe University Human Ethics Committee (approval number 11-052), and consent from the children’s parents was obtained according to the Declaration of Helsinki (BMJ 1991; 302: 1194).

### Apparatus

A Tobii 120 binocular eye tracker and Tobii Studio software (version 3.0.3 Tobii, Stockholm, Sweden) were used to present stimuli and record visual attention and pupil diameter. This system presents stimuli on a computer-like monitor and does not require any equipment to be fastened onto the participant. Using multiple sensors, with bright and dark pupil tracking, a 3D model of the pupil (taking into account optical distortions from the cornea and lens) is built, allowing for both pupil diameter and distance from the screen to be measured at a sampling rate of 60 Hz (one sample every 16.67 ms). With this tracking technique, movement-related artefacts are handled in two ways. Firstly, as pupil diameter is a function of distance from the screen (of participant’s head to the monitor), the effect of head movements perpendicular to the monitor are eliminated from the measure of pupil size on a sample-to-sample basis, using basic principles of trigonometry. Secondly, other head movements (i.e. those parallel to the monitor) are accurately tracked (up to 25 cm per second). Artefacts due to partial head turns and blinks were removed through data reduction algorithms (see ‘Data reduction’ section). The eye-tracking monitor (TFT-LCD; W: 34 cm × H: 27 cm) has a refresh rate of 60 Hz. Brightness was set to 100 %.

### Materials

**Fig. 1:**
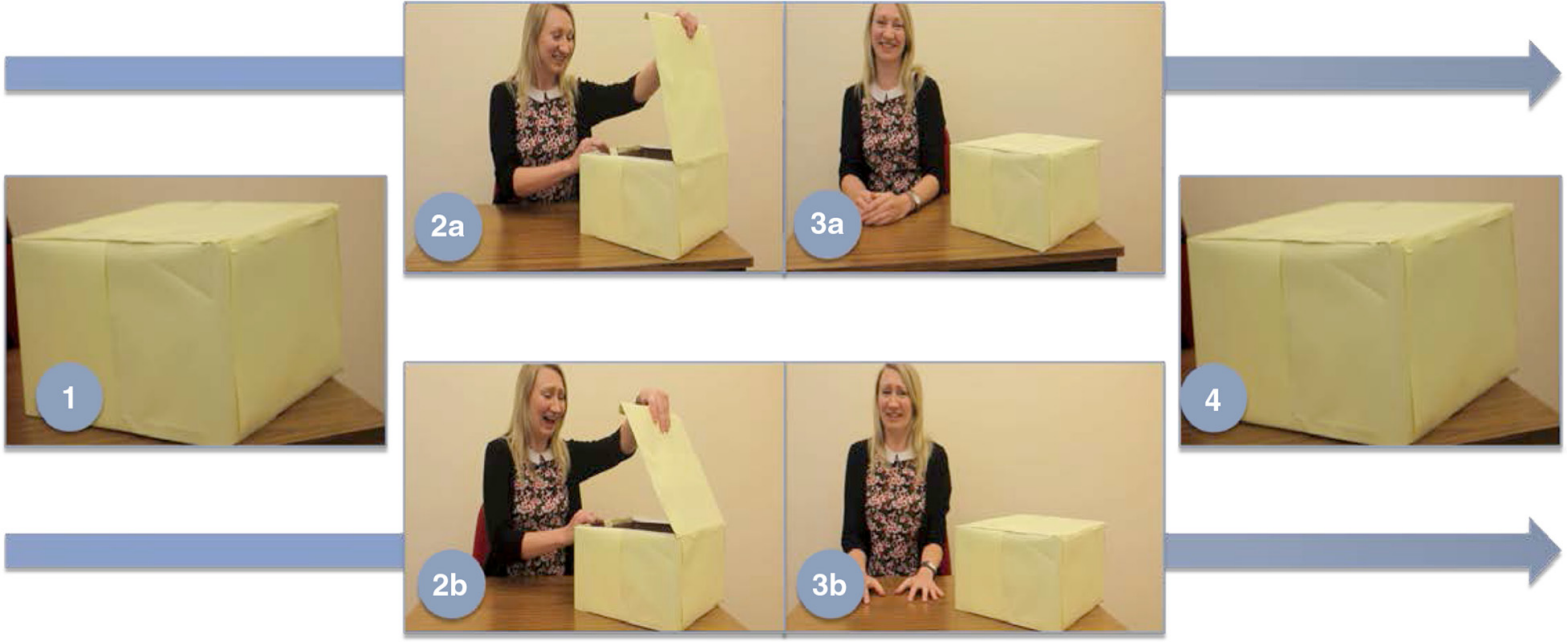
Summary of video sections. *1* Pre-box (box shown before actor’s emotional reactions), *2a/3a* actor reacting happily to contents of box, *2b/3b* actor reacting fearfully to contents of box, *4* post-box (box shown after actor’s emotional reactions). The pre- and post-box are visually identical but are perceived as different (either containing a threatening or positive stimulus) only if the child learns about the contents of the box from the actor’s emotional reactions

There were two study conditions: the happy condition and the fear condition. One video was shown for each of the conditions. Each featured a woman opening a plain box and reacting emotionally (either happily or fearfully) to the occluded object inside. An up-close image of the box was shown *before* and *after* the scene; during these two stimuli, the pupil diameter measure was taken (see Fig. 1 for a summary of the video sections).

First, a scrambled image of the close-up of the box was shown to minimise pre-stimuli to stimulus changes in luminosity (1 s). Second, a close-up still image of the box was shown, the ‘pre-box’ (4 s), before the actor sequence. Third, the video frame zooms from the close-up image of the pre-box to the upper torso of the actor sitting at a table with a neutral face and averted eye gaze, still showing the box in the corner of the frame (happy condition: 7.60 s, fear condition: 8.23 s). Fourth, the actor looks toward the camera with a neutral expression, then looks at the box and moves her arms to open it (happy condition: 5.93 s, fear condition: 5.83 s). Fifth, the actor opens the box and reacts emotionally (happily or fearfully) to the contents of the box, which cannot be seen by the participant. Sixth, the actor closes the box and looks back at the camera, retaining her emotional expression. In total, the emotional expression of the actor is shown for 6.78 s in the happy condition and 6.99 s in the fear condition. Seventh, the camera moves away from the actor’s face and slowly zooms back to a close-up of the box (4.097 s in the happy condition and 4.107 s in the fear condition), to allow enough time for resolution of the pupil dilation associated with viewing an emotional facial expression [53]. Finally, the box is shown again, the ‘post-box’ (4 s). In each of the videos, the still image of the post-box was visually identical to the pre-box. The total video length was 33.41 in the happy condition and 34.15 in the fear condition.

We reasoned that participants would perceive the pre-box as an emotionally neutral stimulus and the post-box as a threatening stimulus (in the fear condition) or a positive stimulus (in the happy condition), as a consequence of calibrating their emotional reactions about the box to those of the actor. Following this line of reasoning, if participants failed to either look or calibrate to the actor’s emotions, then they would continue to perceive the box as a neutral stimulus. The process of change from perceiving the box as a neutral stimulus to perceiving it as an emotionally relevant stimulus was measured by pupil size increase, an index of emotional reactivity. A larger pupil size during viewing of the post-box (an emotionally conditioned stimulus) compared to pre-box (a neutral stimulus) was used as an index of social-emotional calibration.

### Procedure

Testing took place in a well-lit room of the community childcare centre which had no external light. Ambient luminosity was checked prior to each testing session, using a handheld photometer (model PLMX, Quantam Instruments). Ambient luminosity (lux) did not differ during testing between the ASD group (*M =* 25.630, SD *=* .156) and TD group (*M =* 25.628, SD *=* .200), *t*(36) = −.038, *p* = .970. The child was seated in a comfortable chair, approximately 60 cm (36.46° visual angle) from the eye-tracking monitor. The experimenter first calibrated the child’s eye movements with the built-in five-point Tobii Studio calibration procedure. Following this, each child passively viewed the images (with the emotion shown in the first video counterbalanced within each participant group), which were interspersed between the presentation of ‘filler’ stimuli (child-friendly pictures) to maintain attention [61]. The experiment presented here was a part of a larger study examining emotional responses in children with ASD (see [30, 46, 51, 53]).

### Data reduction

Pupil data, preprocessed to minimise large movement artefacts (see ‘Apparatus’ section), were further processed with a custom-built LabVIEW 2010 (National Instruments, Austin, Texas, USA) algorithm (Beaton, unpublished), based on previously published methodology (e.g. [62, 63]) to further screen out movement-related artefacts (including partial head turns and blinks). First, samples for which only one eye was tracked were eliminated (to minimise pupil size miscalculation due to head angle or ambient light exposure). Where both eyes were tracked, a mean pupil diameter across eyes was computed. Second, to remove extreme sample-to-sample changes in pupil diameter due to partial eyelid closures (common in samples either side of missing data due to blinks), samples outside 2 × standard deviations of the mean rate of change (calculated for each participant) were removed. After partial head turn- and blink-related artefacts were deleted, missing pupil data rates were calculated by group (pre-interpolation, whole video): happy condition: ASD group *range =* 1–70 %, *M =* 34 %, SD *=* 21 %, TD group *range =* 2–72 %, *M =* 24 %, SD *=* 20 %; fear condition: ASD group *range =* 2–77 %, *M =* 41 %, SD *=* 25 %, TD group *range =* 2–48 %, *M =* 22 %, SD *=* 15 %. Third, gaps in data, due to blinks, were only linearly interpolated between stable data points (traces) to a maximum of 350 ms [64, 65]. A trace was deemed stable if there were a minimum of 50 % of the samples in 2 × total length of the gap, pre- and post-gap. This method allowed for a differential threshold for linear interpolation, based on gap length and the reliability of the pre/post-gap data.

A relative percentage change measure of pupil dilation (increase in size) was calculated using the last 300 ms of the 1 s scrambled image (to avoid the pupillary light reflex; [66]) which appeared directly before the onset of the pre-box, as a baseline. The following formula was used:

$$ a=\left(b-c\right)/c\times 100 $$

where *a* is the percentage change from baseline to the following sections of the video: pre-box, emotional reaction, zoom in and post-box, *b* is the mean pupil diameter during pre-box, emotional reaction, zoom in and post-box video sections and *c* is the mean pupil diameter during the 300 ms before the onset of the pre-box (i.e. the last 300 ms of the scrambled image), per participant. To create a variable to represent social-emotional calibration we then subtracted the new relative pre-box variable from the post-box variable, where positive values equals more social-emotional calibration. As the pre- and post-box was visually identical, more pupil dilation in the post- vs. pre-box was taken as an index of learning about the happy- or fear-inducing contents of the box, through the actors’ emotional expressions shown in the scene.

**Fig. 2:**
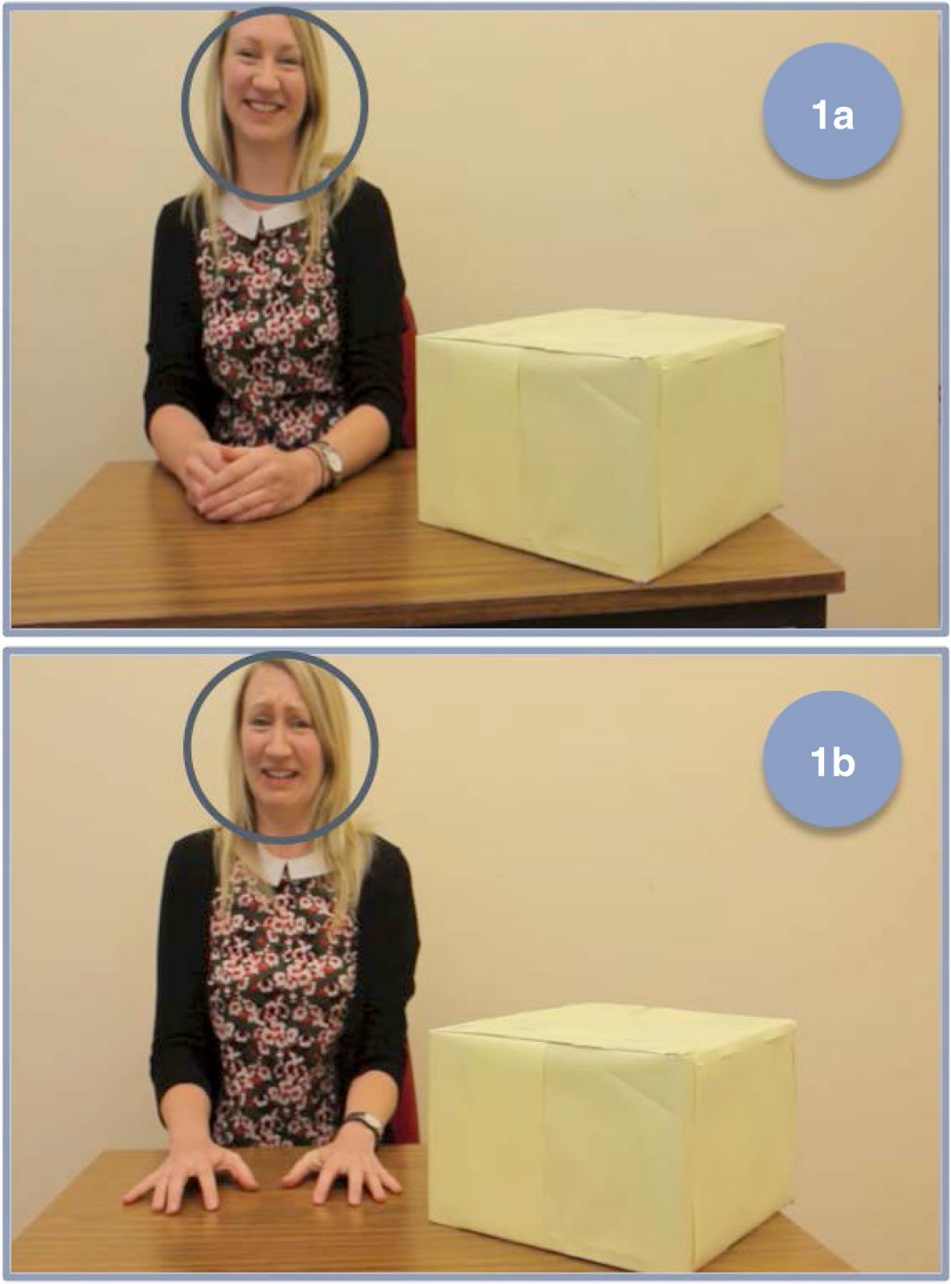
Face areas of interest (AOIs) for the happy (*1a*) and fear (*1b*) conditions. These AOIs last the total time the emotional expressions are shown on the actor’s face (happy condition = 6.78 s, fear condition = 6.99 s), from when she reacts after opening the box to before the camera zooms back into the box close-up

Two sets of areas of interest (AOIs) were created with the Tobii Studio software. The first was for the happy and fearful faces to measure visual attention to these facial expressions (see Fig. 2) and the second was to measure visual attention to the pre- and post-boxes (these were the size of the whole screen). Visual attention data (total fixation duration within the face AOIs) was also extracted from Tobii Studio using a fixation filter (I-VT), using the default pre-sets (maximum gap length 75 ms, window length 20 ms, velocity threshold 30 degrees per second, maximum time between fixations 75 ms, maximum angle between fixations .5°), with the exception that the minimum fixation duration was set to 100 ms. This minimum fixation duration was chosen as eye-tracking data of 100 ms or more are not only more reliable than data tracked for shorter durations [67] but are also considered to be a reliable index of what elements in a scene are actually captured and processed [68].

## Results

Data were first analysed for skewness, kurtosis and outliers using the method outlined in [69], with a critical value set at +/−3.29. Data were normally distributed (all critical values under 3.18); therefore, parametric tests were used in all analyses. As the index of social-emotional calibration (pre- to post-box pupil dilation) in the two emotion conditions (happy, fear) was not correlated in the TD group (*r* = −09, *p* = .69), and only marginally correlated in the ASD group (*r* = .45, *p* = .05), the happy and fear conditions were analysed separately.

### Visual attention to the pre- and post-boxes

To determine whether the groups differed in their visual attention to the pre- and post-box images, independent sample *t* tests were performed on the total fixation durations within the AOIs for the up-close images of the box which appeared before and after the actor scene (4 s each). The groups differed in visual attention on the happy condition pre-box (*t*(38) = 2.66, *p =* .01) and the fear condition post-box (*t*(38) = 3.81, *p <* .001), but not the happy condition post-box (*t*(38) = 1.41, *p =* .17) or the fear condition pre-box (*t*(38) = 1.91, *p =* .06). However, both groups viewed each of the boxes long enough for a stable measure of pupil diameter [67, 68] (TD happy pre- and post-box: *M =* 2.62 s, SD *=* .82 s and *M =* 2.24 s, SD *=* .58 s, respectively; ASD happy pre- and post-box: *M =* 1.94 s, SD *=* .81 s and *M =* 1.91 s, SD *=* .86 s, respectively; TD fear pre- and post-box: *M =* 2.52 s, SD *=* .54 s and *M =* 2.76 s, SD *=* .76 s, respectively; ASD fear pre- and post-box: *M =* 2.11 s, SD *=* .78 s and *M =* 1.80s, SD *=* .83 s, respectively).

### Visual attention to the emotional expressions

**Fig. 3:**
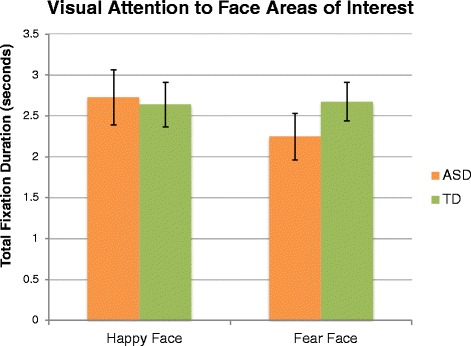
Visual attention to the emotional expressions. Total fixation duration (sum of individual fixation durations) to the emotional facial expression area of interest for the happy and fear social-emotional calibration videos, for each group. *Error bars* represent standard error of the mean

To determine whether the groups differed in their visual attention toward the emotional expressions of the actor, two independent sample *t* tests were performed on the total fixation durations within the AOIs for the fear and happy expression for all frames in which the expression was shown in the video. For both the happy and fear AOIs, there were no group differences in visual attention (*t*(38) = −.21, *p =* .84 and *t*(38) = 1.16, *p =* .26, respectively). Means for total fixation duration within the happy and fear face AOIs are presented in Fig. 3.

### Social-emotional calibration: pupil dilation from pre- to post-box

To determine whether the groups showed social-emotional calibration to the actor, we compared the percentage pupil change from baseline in the pre- and post-box. We conducted two 2 groups (ASD, TD) × 2 boxes (pre, post), repeated measures ANCOVAs, one each for the fear and happy conditions. The dependent variable was mean percentage pupil dilation from baseline, with cognitive ability (MSEL composite score) as a covariate. As the effect of the cognitive ability covariate was not significant for either happy, *F*(1,37) = 1.25, *p =*.27, *η*^2^ *=* .03, or fear, *F*(1,37) = .003, *p =* .97, *η*^2^ *<* .001, both analyses were re-run without this covariate. For both the happy and fear ANOVAs, the group main effect was not significant (*F*(1,38) < .001, *p =* .99, *η*^2^ *<* .001 and *F*(1,38) = .87, *p =* .36, *η*^2^ *=* .02, respectively), but the box main effect was (*F*(1,38) = 23.78, *p <* .001, *η*^2^ *=* .38 and *F*(1,38) = 16.90, *p <* .001, *η*^2^ *=* .31, respectively). These effects were both driven by box × group interactions, with the happy condition significant (*F*(1,38) = 9.42, *p =* .004, *η*^2^ *=* .20) and the fear condition marginally significant (*F*(1,38) = 2.85, *p =* .09, *η*^2^ *=* .07, respectively).

**Fig. 4:**
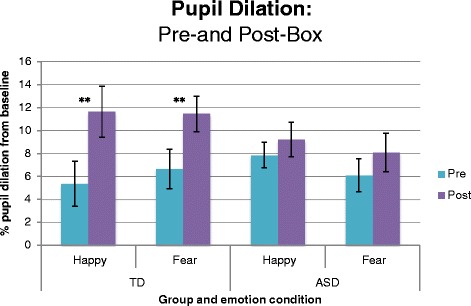
Percentage dilation from baseline in each emotion condition for the pre- and post-box. *Error bars* represent standard error of the mean. **Pairwise comparisons (Bonferroni-corrected) show difference pre- to post-box in the TD group only, for happy (*p <* .001, *η* ^2^ *=* .45) and fear (*p <* .001, *η* ^2^ *=* .31). *Error bars* represent standard error of the mean

**Fig. 5:**
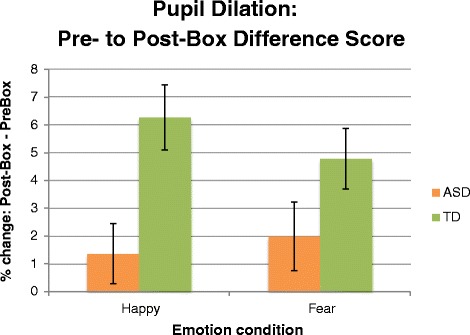
Post-minus pre-box difference score as an index of social-emotional calibration. Higher scores = more social-emotional calibration. *Error bars* represent standard error of the mean

Follow-up pairwise comparisons (Bonferroni-corrected) showed that whilst the TD group had a larger mean pupil diameter during the post- vs. pre-box for happy *F*(1,38) = 30.74, *p <* .001, *η*^2^ *=* .45, and fear *F*(1,38) = 16.82, *p <* .001, *η*^2^ *=* .31, the ASD group did not show a mean pupil size change of this magnitude in either the happy *F*(1,38) = 1.45, *p =* .24, *η*^2^ *=* .04 or the fear condition *F*(1,38) = 2.93, *p =* .10, *η*^2^ *=* .07. Mean percentage pupil dilation from baseline in each condition is presented in Fig. 4, and the difference scores between the pre- and post-box conditions are presented in Fig. 5.

### Reaction to actor’s emotional expressions

**Fig. 6:**
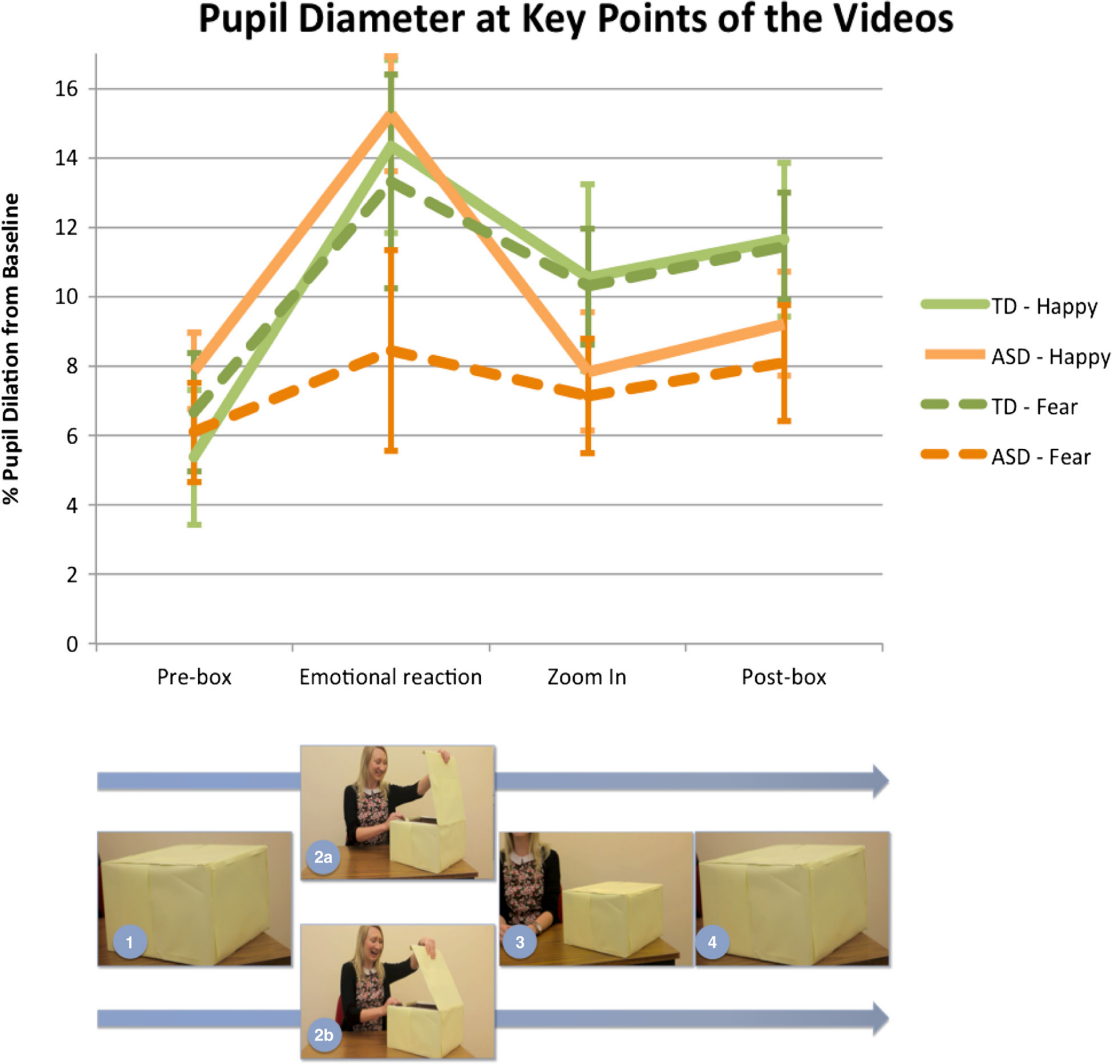
Mean pupil diameter across key phases of the video, pre-box (the first presentation of the box, before the emotional reaction), emotional reaction, zoom in (during which camera zooms in the post-box) and post-box (the second presentation of the box, after the emotional reaction). *Error bars* represent standard error of the mean

To examine whether the groups differed in their pupil dilation to the actor’s emotional reaction in each of the videos, independent sample *t* tests were performed on mean percentage pupil dilation from baseline to the emotional reaction of the actor, which was displayed in the video from 18:53 to 25:31 in the happy condition and 19:06 to 26:05 in the fear condition. Mean percentage pupil dilation from baseline to the pre-box, emotional reaction and post-box phases of the video are presented in Fig. 6. As the pupil dilation in the post-box may have included prolonged resolution of pupil dilation from the emotional reaction phase, the zoom in section was also included in the figure. Though the group means appeared different on the fear condition, variability within group was large (ASD: *M* = 8.45, SD = 12.99; TD: *M* = 13.32, SD = 13.80), so the groups did not differ significantly in their pupil responses to the actor’s happy on this condition (*t*(38) = 1.15, *p =* .26) nor the happy condition (*t*(38) = −31, *p =* .76; ASD: *M* = 15.27, SD = 7.44; TD: *M* = 14.33, SD = 11.15).

### Associations of social-emotional calibration with ASD symptoms

**Fig. 7:**
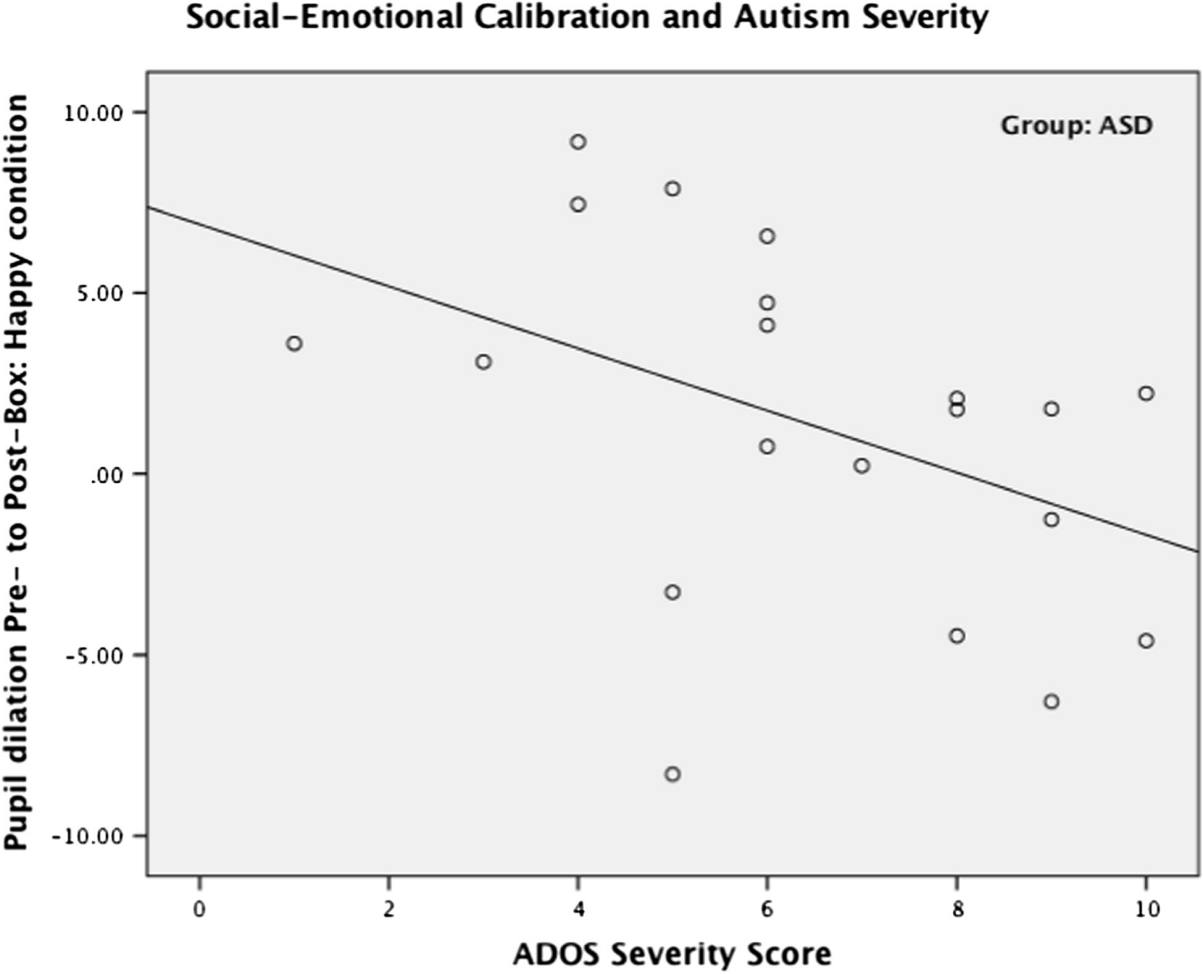
Scatterplot with social-emotional calibration index on the *Y* axis and the ADOS Severity Score on the *X* axis, showing a moderate negative correlation (*r* = −.43, *p* = .03)

To explore whether social-emotional calibration was related to ASD symptoms, we ran Pearson correlations between the calibrated ADOS scores (Social Affect scale, Restricted and Repetitive Behaviors scale and Severity Score) and the post-minus pre-box difference score (social-emotional calibration index) on the happy and fear conditions. Positive values index more social-emotional calibration. A moderate negative correlation was found between the Severity Score and the social-emotional calibration index on the happy condition (*r* = −43, *p* = .03; see Fig. 7), and there was a trend of negative association between the Restricted and Repetitive Behaviors scale and the social-emotional calibration index on the fear condition (*r* = −.36, *p* = .06). No other correlations were significant (Social Affect scale with happy and fear conditions: *r* = −.21, *p* = .19 and *r* = .24, *p* = .15, respectively; Restricted and Repetitive Behaviors scale with happy condition: *r* = −.29, *p* = .11; Severity Score with fear condition: *r* = −.11, *p* = .33).

## Discussion

The aim in this study was to examine whether young children with ASD show calibration to others’ emotions. Through the analysis of pupillary reactions, we found that TD children but not children with ASD learned about the emotional value of objects via vicarious emotional reactions. This was not due to a lack of attention or a reduced emotional response to the expressions of the actor; the children with and without ASD did not differ in their visual attention or concurrent pupil dilation to the actor’s facial expressions. Thus, results are consistent with the notion of reduced social-emotional calibration in preschoolers with ASD. Interestingly, calibrating to the actor’s happy expressions, but not to the fear expressions, was associated with overall ASD severity. This result suggests that autism characteristics are related to the extent to which a person with ASD can quickly internalise and align to the positive emotions of those around them regarding features of their environment. Further research is needed to substantiate this interpretation.

A somewhat surprising finding in the current study was that children with ASD did not differ from their TD peers in their attention to the actor’s face. This finding, whilst inconsistent with early eye-tracking research (e.g. [20]), accords with numerous recent findings suggesting normative patterns of visual attention in response to social stimuli in ASD under specific circumstances (e.g. [53, 70–72]). Likewise, reduced concurrent response to the actor’s emotions may have been expected given findings of reduced emotional reactivity to others in ASD. However, our finding is consistent with some studies showing no group differences in emotional responses to others during longer presentations of emotions [3, 30].

It is important to consider not only both the mechanisms which drive difficulties in social-emotional calibration in ASD but also the effects that difficulties in this area have on other areas of social development and behaviour in ASD. Documented difficulties in dyadic emotional reactivity, emotion recognition and/or in triadic/joint attention in ASD [3, 73–76] are likely to impact social-emotional calibration in this population, and more so than associative learning mechanisms, which are thought to be intact in ASD (e.g. [77]). Further research is now needed to confirm the foundation skills required for social-emotional calibration.

The finding of an association between ASD symptoms and social-emotional calibration may help to explain the idiosyncratic responses individuals with this disorder characteristically have to people and objects. Individuals with ASD also commonly present with idiosyncratic emotional reactions to sensory features of the environment; one hypothesis is that these arise from difficulties calibrating to others’ emotional states about these sensations, i.e. to the *neutral* emotional states during everyday activities. Common everyday activities can cause extreme emotional reactions to children with ASD (e.g. vacuuming, having a haircut, rearranging home furniture and doing the grocery shopping), leading to meltdowns and impairing family functioning [7, 78]. Thus, future research should examine in more detail the relationship between social-emotional calibration and atypical emotional reactions to sensory features of the environment in ASD and between social-emotional calibration and emotion regulation in this population. This result has important implications for intervention strategies and targets, providing a case for using therapeutic styles which capitalise on positive-affect joint attention frameworks for teaching skills [79–81] and explicitly teaching social-emotional calibration to positive events, objects and other experiences.

A deficit in social-emotional calibration arguably has important implications for social and cultural development. Aligning to others’ emotions in a myriad of contexts, throughout development, teaches children about their world and the objects, events and people in it, including the cultural practices, habits and attitudes of a particular society. These everyday learning opportunities provide children with rich information to guide their behaviour, judgements and attitudes. A deficit in calibrating to others’ emotion/s regarding features of the environment, as documented in this study in children with ASD, is likely to negatively impact learning from their social environment and their participation in the cultural and sub-cultural practices of the societies in which they live.

As social-emotional calibration may be characterised as an implicit learning process, difficulties in calibrating to others’ emotions may be a result of difficulties in implicit learning in ASD and so this matter should be investigated further. Furthermore, as the paradigm in the current study was essentially a one-trial learning paradigm, further work is needed to understand whether people with ASD do calibrate to others’ emotions about features of the environment following multiple presentations. Other factors which affect social learning (such as temperament and social closeness to the target person) are likely to have an impact on social-emotional calibration and should also be investigated in future work. In addition to physiological reactivity indices of social-emotional calibration, another future direction would be to investigate changes in behavioural patterns that may occur after calibrating to one’s emotions.

### Limitations

Whilst the inclusion of an age-matched TD group afforded an understanding of normative reactivity, it is important to acknowledge that the two groups were not matched on cognitive ability. The inclusion of a chronological- and mental age-matched group would have been ideal, and further research should seek to incorporate such a control group. Nonetheless, it should be noted that the cognitive ability covariate was not significant in the analysis.

As the children with ASD in the current study were mostly low functioning with significantly delayed receptive language ability, it was not possible to administer an emotion recognition task to understand the contribution of emotion recognition difficulties to diminished social-emotional calibration. Future research should aim to include such a task, only possible with more able children, to clarify this issue.

Although physiological measures can provide insight into the emotion processing of lower-functioning individuals, physiological measures do not distinguish valence of one’s emotional reaction (i.e. whether viewing happy expressions makes oneself feel happy), rather they provide information on emotional arousal. Therefore, future research on social-emotional calibration should incorporate measures, such as behavioural coding of facial affect, to provide information on the valence of emotional reactions and how these relate to aligning with others’ behaviour.

## Conclusions

The current study explored whether children with ASD calibrate to others’ emotional expressions about features of their environment, an aspect of social learning in ASD that has received little empirical attention to date. The pupil dilation results are consistent with the presence of social-emotional calibration in TD children, but not in children with ASD. By studying this process of social-emotional calibration, we can better understand the difficulties people with ASD have in learning from others and participating in cultural and sub-cultural practices, as well as the idiosyncratic responses that are characteristic of this population. Further work is needed to substantiate this interpretation and understand the mechanisms that underlie social-emotional calibration and the effects of this phenomenon on social development.

## Abbreviations

ADOS, Autism Diagnostic Observation Schedule; AOI, area of interest; ASD, autism spectrum disorder; ECG, electrocardiogram; ERP, event-related potentials; fMRI, functional magnetic resonance imaging; MSEL, Mullen Scales of Early Learning; TD, typically developing
